# A117 MANAGEMENT OF VARICEAL BLEEDING WITH ENDOSCOPIC INJECTION OF N-BUTYL-2-CYANOACRYLATE IN PREGNANCY

**DOI:** 10.1093/jcag/gwab049.116

**Published:** 2022-02-21

**Authors:** S Vukovic, Z Gallinger

**Affiliations:** 1 Internal Medicine, University of Toronto Temerty Faculty of Medicine, Toronto, ON, Canada; 2 Mount Sinai Hospital Schwartz/Reisman Emergency Centre, Toronto, ON, Canada

## Abstract

**Background:**

Although variceal bleeding is rare in pregnancy, pregnant woman with non-cirrhotic portal hypertension are at greater risk of variceal hemorrhage due to increased splanchnic blood flow. Variceal hemorrhage is associated with increased risk of maternal and fetal mortality. Most documented cases of variceal bleeding in pregnancy report the use of elastic ligation for hemostatic management. Histoacryl glue has become increasingly utilized in the acute management and secondary prophylaxis of gastric and ectopic varices in the general population.

**Aims:**

Evidence surrounding the use of Histoacryl glue in pregnancy is limited. We report two cases of variceal bleeding during pregnancy managed successfully with glue.

**Methods:**

Varices were confirmed endoscopically. Images were obtained and reviewed. Aliquots of injected glue were recorded. Maternal and fetal outcomes were monitored during pregnancy and post-partum.

**Results:**

*Case 1*: A 39-year-old G4P3L0, presented at 20 weeks gestation following several episodes of hematemesis. Urgent esophagogastroduodenoscopy (EGD) revealed hyperemia and portal hypertensive gastropathy in the fundus. A gastric varix with high-risk stigmata was injected with a total of 9mL of 50/50 Histoacryl/Lipodol and bleeding stopped. The patient returned to the hospital two weeks following discharge with melena. Repeat EGD showed mild portal hypertensive gastropathy with a clot at the site of the previous bleeding. No further intervention was required, and the patient delivered at 37 weeks via elective caesarian-section.

*Case 2:* A 41-year-old G2P0L1 presented at 26 weeks gestation with severe anemia refractory to five units of blood transfusion. Urgent EGD showed a large gastric varix with recent stigmata of hemorrhage, but no active bleed. A total of 13 mL of 75/50 Histoacryl/Lipidol was injected into the varix with no further bleeding. She delivered by elective caesarian-section at 37 weeks gestation. Repeat EGD performed following delivery showed a larger varix with fresh clot at the inferior portion of the fundus. Due to concern for re-bleeding, a total of 4 mL of 50/50 Histoacryl/Lipidol was injected at the site. CT scan post-partum showed prominent gastric varices with glue visualized within the peripheral branches of the portal vein (Figure 1).

**Conclusions:**

Our cases highlight the successful management of gastric varices using Histoacryl glue in pregnancy. Risks associated with Histoacryl glue include glue embolization, local venous thrombosis, as well as extrusion of glue and ulceration. The general complication rate is quoted between 0.5%-5%. One of the two cases was associated with a maternal complication, with evidence of new portal vein thrombosis following injection. The safety of sclerotherapy in pregnancy with cyanoacrylate remains unclear, and further evaluation of the management of gastric varices in this unique population are required.

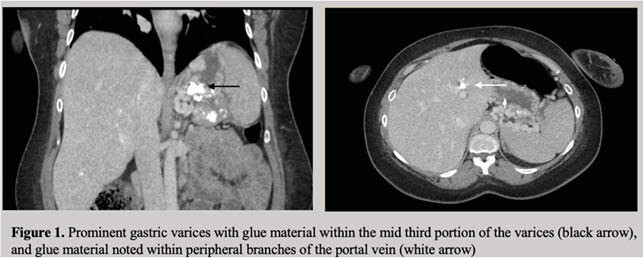

**Funding Agencies:**

None

